# Enteric Fever and Invasive Nontyphoidal Salmonellosis—9th International Conference on Typhoid and Invasive NTS Disease, Bali, Indonesia, April 30–May 3, 2015

**DOI:** 10.3201/eid2204.151463

**Published:** 2016-04

**Authors:** Mohammed Imran Khan, Cara Katrinak, Alexander Freeman, Carlos Franco-Paredes

**Affiliations:** Aga Khan University, Karachi, Pakistan (M. Imran Khan);; Coalition against Typhoid–Sabin Vaccine Institute, Washington, DC, USA (C. Katrinak, A. Freeman);; Hospital Infantil de México Federico Gómez, Mexico City, Mexico (C. Franco-Paredes)

**Keywords:** enteric fever, typhoid fever, salmonella infections, nontyphoidal salmonellosis, enterobacterial infections, bacteria, conferences, Indonesia

Invasive salmonellosis causes a spectrum of diseases, including enteric fever (i.e., typhoid and paratyphoid fever) and nontyphoidal *Salmonella* (NTS) infection (*1*). This group of bacterial pathogens continues to inflict a large burden of disease globally, especially in countries with low and middle incomes (*2*). No global control strategy yet exists because of multiple challenges, such as low availability of effective vaccines, poor access to clean water and sanitation, lack of regional or national data, and difficulties in scaling up interventions. Recent progress in vaccine development, technology transfer, and disease surveillance has increased prospects of typhoid fever control and eventual control of paratyphoid and NTS.

In response to these issues, the 9th International Conference on Typhoid and Invasive NTS Disease took place in Bali, Indonesia, during April 30–May 3, 2015 (http://www.coalitionagainsttyphoid.org/typhoid-conference/conference-history/9th-conference-resources/). The Coalition against Typhoid Secretariat, based at the Sabin Vaccine Institute (Washington, DC, USA), partnered with PT Bio Farma, a vaccine manufacturer (Bandung, Indonesia), to convene ≈250 participants from 36 countries at a 4-day event sponsored by the Bill & Melinda Gates Foundation (Seattle, WA, USA). Conference objectives were to inform the audience about progress made in the epidemiologic understanding of enteric fever and invasive NTS (iNTS) disease, in advancements in diagnostics and clinical management, and in the development and use of vaccines to prevent the diseases.

## Epidemiology, Pathogenesis, and the Burden of Enteric Fever and iNTS

On the basis of preliminary findings of recent research presented by experts at the conference, enteric fever and iNTS disease are major public health problems in Africa, particularly among children (*3*). A study from the Pacific region highlighted behavioral, infrastructural, and environmental risk factors for typhoid fever, such as sanitation practices, water supply infrastructure, and flooding (*4*). The environmental, sociocultural, and behavioral determinants of typhoid highlighted in a study in Fiji suggest that cases are more likely to occur in rural than in urban areas (*5*); results from a similar study in Nepal indicate that enteric fever is predominantly an urban and periurban disease (*6*). However, both studies agree that typhoid cases are likely to occur in areas with compromised water and sanitation facilities and at low elevations. Genetic research suggests that the HLA-DRB1*0405 gene causes a 5-fold decrease in typhoid fever risk (*7*). The recently discovered typhoid toxin, which is encoded by *Salmonella enterica*, leads to cell cycle arrest in target cells (*8*). The toxin binds Neu5Ac-terminated glycans, which are expressed exclusively in human cells, resulting in the pathogen’s host specificity.

A phylogenetic analysis of *S. enterica* subspecies *enterica* serovar Typhi/*S. enterica* ser. Paratyphi A lineages found that *S.* *enterica* ser. Typhi haplotype H58 is a multidrug-resistant lineage that has become dominant in the last 20 years (*9*). This strain could explain the emergence of increased typhoid fever incidence in certain regions around the world. Beginning in 1999, Malawi had outbreaks of *S. enterica* ser. Enteriditis*, S. enterica* ser. Typhimurium *ST313*, and *S. enterica* ser. Typhi, all of which were related to emerging multidrug-resistant strains (*10*). A study on *S. enterica* ser. Typhi and *S. enterica* ser. Paratyphi A showed high resistance to nalidixic acid and ciprofloxacin; Nepal’s National Antibiotic Treatment Guideline (http://www.mohp.gov.np/index.php/publication/155-national-antibiotic-treatment-guideline) recommends ciproflaxacin for treatment of enteric fever, highlighting the need for updated treatment guidelines (*11*). Continued testing of *S. enterica* ser. Typhi strains for drug resistance will provide further evidence that treatment is becoming more difficult, leading to increased incidence of complicated cases. Data from febrile patients admitted to Om Hospital and Research Center in Nepal indicate that *S. enterica* ser. Paratyphi A appears to cause enteric fever more often than does *S. enterica* ser. Typhi (*12*). *S. enterica* ser. Paratyphi A produces a milder infection than the other serotype but is frequently associated with a lengthened fever clearance time because of a higher minimum inhibitory concentration to all tested antimicrobial drugs (*13*).

Research on the relationships between typhoid or iNTS disease and other co-infections (e.g., HIV or malaria) was also a highlight of the conference. The spread of *S. enterica* ser. Typhimurium in sub-Saharan Africa was associated with the spread of HIV (*14*) and malaria (*15*). Additional risk factors for iNTS disease include sickle-cell disease, schistosomiasis, malnutrition, and immunosuppression (*16*). Presenters echoed a need for improved diagnostics for enteric fever and iNTS; most cases in areas to which these diseases are endemic are reported without microbiologic confirmation. Preliminary studies indicate that a quantitative real-time PCR developed by Fondation-Mérieux (Lyon, France) shows promise for diagnostic testing (*17*).

## Prevention Strategies

Since the previous international conference in 2013, at least 6 additional conjugate vaccines for typhoid fever are in various phases of clinical development, and another has been licensed in India. The new conjugate vaccines are immunogenic in infants >6 months of age and offer a longer duration of protection, compared with existing Vi polysaccharide vaccines (*18*). A bivalent conjugate vaccine against *S.* *enterica* serovars Enteritidis and Typhimurium is also being developed, along with several *S. enterica* ser. Paratyphi A conjugate vaccines associated with O-antigen, including bivalent *S. enterica* serovars Typhi and Paratyphi A vaccines (*19*).

Although vaccination against enteric fever and iNTS remains a priority, many countries have eliminated typhoid fever without using vaccines. Research from India showed the role of water sanitation improvements in decreasing incidence of typhoid fever. In this study, continuous water supply was associated with a 42% decrease in reported typhoid and a 22% decrease in bloody diarrhea (*20*). Several studies showed that typhoid is more likely to strike areas close to compromised water and sanitation facilities, indicating that water, sanitation, and hygiene interventions should synergize efforts to control enteric fever. These studies focus on typhoid; additional data are needed to inform efforts in nonvaccine control of iNTS disease.

In conjunction with the conference, the Coalition against Typhoid published a supplement on vaccines to provide further information about enteric fever control (*21*). Although the advances and achievements presented at this conference are promising, a global control strategy is urgently needed that will bring together scientists, vaccine manufacturers, public health agencies, and policy makers.
